# Late Failure of High-Flow Nasal Cannula May Be Associated with High Mortality in COVID-19 Patients: A Multicenter Retrospective Study in the Republic of Korea

**DOI:** 10.3390/jpm11100989

**Published:** 2021-09-30

**Authors:** Ae-Rin Baek, Gil Myeong Seong, Song-I Lee, Won-Young Kim, Yong Sub Na, Jin Hyoung Kim, Bo Young Lee, Moon Seong Baek

**Affiliations:** 1 Division of Allergy and Pulmonology, Department of Internal Medicine, Soonchunhyang University Bucheon Hospital, Bucheon 14584, Korea; aerinbaek@gmail.com; 2 Department of Internal Medicine, Jeju National University College of Medicine, Jeju 63243, Korea; rolland0211@gmail.com; 3 Department of Pulmonary and Critical Care Medicine, Chungnam National University Hospital, Daejeon 35015, Korea; newcomet01@naver.com; 4 Department of Internal Medicine, Chung-Ang University Hospital, Chung-Ang University College of Medicine, Seoul 06973, Korea; wykim81@cau.ac.kr; 5 Department of Pulmonology and Critical Care Medicine, Chosun University Hospital, Gwangju 61453, Korea; ebusters@chosun.ac.kr; 6 Division of Respiratory and Critical Care Medicine, Department of Internal Medicine, Ulsan University Hospital, University of Ulsan College of Medicine, Ulsan 44033, Korea; firebomb@daum.net; 7 Division of Allergy and Respiratory Diseases, Soonchunhyang University Seoul Hospital, Seoul 04401, Korea; etboss2@gmail.com

**Keywords:** COVID-19, high-flow nasal cannula, ROX index, intubation, mortality

## Abstract

The aim of this study was to determine whether the late failure of high-flow nasal cannula (HFNC) is associated with mortality in patients with coronavirus disease 2019 (COVID-19). This multicenter study included seven university-affiliated hospitals in the Republic of Korea. We collected the data of patients hospitalized with COVID-19 between 10 February 2020 and 28 February 2021. Failure of HFNC was defined as the need for mechanical ventilation despite HFNC application. According to the time of intubation, HFNC failure was divided into early failure (within 48 h) and late failure (after 48 h). During the study period, 157 patients received HFNC and 133 were eligible. Among them, 70 received mechanical ventilation. The median time from HFNC initiation to intubation of the early failure group was 4.1 h (interquartile range [IQR]: 1.1–13.5 h), and that of the late failure group was 70.9 h (IQR: 54.4–145.4 h). Although the ratio of pulse oximetry/fraction of inspired oxygen (ROX index) within 24 h of HFNC initiation tended to be lower in the early failure group than in the late failure group, the ROX index before two hours of intubation was significantly lower in the late failure group (odds ratio [OR], 5.74 [IQR: 4.58–6.98] vs. 4.80 [IQR: 3.67–5.97], *p* = 0.040). The late failure of HFNC may be associated with high mortality in COVID-19 patients with acute respiratory failure.

## 1. Introduction

Coronavirus disease 2019 (COVID-19), caused by severe acute respiratory syndrome coronavirus 2 (SARS-CoV-2), often results in life-threatening conditions such as severe pneumonia with respiratory failure and even death. One week after illness onset, dyspnea can be aggravated and acute respiratory distress syndrome (ARDS) can develop [1]. The World Health Organization published the Clinical Progression Scale to classify the severity of COVID-19 patients, where patients hospitalized for severe disease were divided into use of high-flow nasal cannula (HFNC) and mechanical ventilation [2]. As such, HFNC and mechanical ventilation are the bases of the treatment of COVID-19 patients with respiratory failure.

HFNC is a non-invasive oxygen delivery device, which can supply constant FiO_2_ up to 100%, with a maximum flow of 60 L/min [3,4]. HFNC supplies heated and humidified air and is used to facilitate easier work for breathing and positive end-expiratory pressure [5]. Furthermore, compared to conventional oxygen therapy, HFNC may reduce the need for invasive mechanical ventilation in the case of hypoxemic respiratory failure (risk ratio [RR], 0.85; 95% CI, 0.74–0.99) [6]. However, there have been concerns about increased mortality due to the delayed intubation of patients after application of HFNC [7,8]. In an observational study of critically ill patients, the overall intensive care unit (ICU) mortality was higher in patients who underwent late intubation than in those who underwent early intubation (39.2% vs. 66.7%, *p* = 0.001) [7].

Mechanical ventilation with protective ventilation strategies is a cornerstone in the treatment of ARDS [9]. According to an observational study involving patients with ARDS, the 60-day mortality was significantly higher in the late intubation group than in the early intubation group (56% vs. 36% *p* < 0.03) [10]. However, whether delayed intubation is related to increased mortality in COVID-19 patients is still debated. We hypothesized that prolonged use of HFNC application is associated with increased mortality because the high respiratory drive of the spontaneous breathing could cause self-inflicted lung injury [11]. The aim of this study was to determine whether late failure of HFNC is associated with increased mortality in COVID-19 patients; furthermore, we determined the risk factors for mortality.

## 2. Materials and Methods

### 2.1. Study Design and Patients

This multicenter retrospective cohort study was conducted at seven university-affiliated referral hospitals in the Republic of Korea. Patients with infection with SARS-CoV-2 confirmed by real-time reverse transcription-polymerase chain reaction analysis were admitted or transferred from the public hospitals for clinical deterioration. Between 10 February 2020 and 28 February 2021, consecutively hospitalized patients (aged over 18 years) treated with oxygen therapy with COVID-19 were enrolled. Patients were excluded if they did not receive HFNC, had a “do-not-intubate” order, or were intubated after HFNC weaning. HFNC as a weaning tool from mechanical ventilation was not considered as HFNC application.

Due to the retrospective nature of the study, the need for informed consent was waived by the Institutional Review Board (IRB) of Chung-Ang University Hospital (2103-009-19360) and the IRBs of the participating hospitals approved the study protocol.

### 2.2. Data Collection and Definitions

The following clinical data were collected from the electronic medical records: age and sex, body mass index, history of smoking, symptoms at admission, time from symptom onset to admission, time from HFNC initiation to intubation, CURB-65, Sequential Organ Failure Assessment (SOFA) score, Acute Physiology and Chronic Health Evaluation (APACHE) II score, Charlson Comorbidity Index (CCI), comorbidities, vital signs at admission, duration of fever, PaO_2_/FiO_2_ at HFNC initiation, laboratory findings, and initial chest radiography findings (normal or unilateral vs. bilateral or multifocal). Data on treatment for COVID-19 and mechanical ventilation (remdesivir, antibiotics, vasopressor, continuous renal replacement therapy, corticosteroid, neuromuscular blocking agent, and prone positioning) were also extracted. Respiratory parameters such as oxygen saturation (SpO_2_), fractional inspired oxygen (FiO_2_), SpO_2_/FiO_2_, and ROX index during HFNC application were collected.

The ROX index is calculated as the ratio of SpO_2_ to FiO_2_ to respiratory rate [12]. CURB-65 is a prediction tool for pneumonia that considers the following factors: Confusion, Urea nitrogen, Respiratory rate, Blood pressure, and age being 65 years or older [13]. Failure of HFNC was defined as escalation of respiratory support to invasive mechanical ventilation despite HFNC application [14,15]. According to the time from HFNC initiation to intubation, HFNC failure was divided into early failure (within 48 h) and late failure (after 48 h) [7]. The primary outcome was in-hospital mortality. Secondary outcomes were length of hospital stay, duration of mechanical ventilation, and tracheostomy.

### 2.3. Statistical Analysis

Continuous variables were reported as median (interquartile range) and categorical variables were specified as number (percentage). The Mann–Whitney U test was used to compare the medians, and Pearson’s chi-square test or Fisher’s exact test were used to compare categorical data between the groups. Univariate analysis and multivariable logistic regression analyses were performed to identify the predictive factors for in-hospital mortality. Multivariable logistic regression analysis was performed using the backward elimination method. Candidate variables for inclusion in the multivariable regression model were the clinically relevant variables with *p*-values < 0.1 in the univariate analysis. Calibration of the logistic regression model was evaluated by the Hosmer–Lemeshow goodness-of-fit test. Discrimination of prediction for in-hospital mortality was determined by receiver operating characteristic (ROC) curve analysis. A two-sided *p*-value of <0.05 was considered statistically significant in all analyses. Statistical analyses were performed using the Statistical Package for the Social Sciences (SPSS) version 26.0 (IBM Corporation, Armonk, NY, USA).

## 3. Results

**Figure 1:**
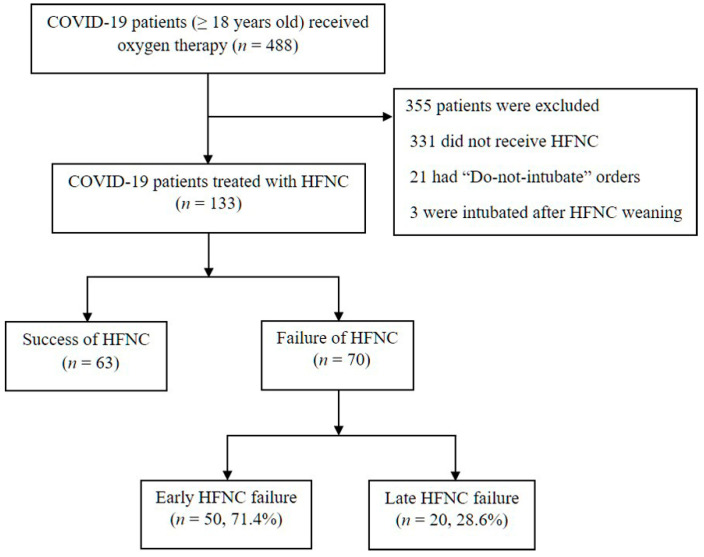
Study flow chart. COVID-19 = coronavirus disease 2019, HFNC = high-flow nasal cannula. Failure of HFNC was defined as the need for mechanical ventilation despite HFNC application: early failure (within 48 h) and late failure (after 48 h).

During the study period, 488 consecutive COVID-19 patients received oxygen therapy at seven hospitals, and 133 treated with HFNC were eligible (331 patients were excluded due to not requiring HFNC, 21 had “do-not-intubate” orders, and 3 were mechanically ventilated after HFNC weaning) (Figure 1). Among the 13,363 were successfully weaned from HFNC, and the remaining 70 mechanically ventilated patients were analyzed. The median time from HFNC initiation to intubation was 11.3 h (IQR: 2.0–46.7 h), and the majority of the patients were intubated within 48 h of HFNC initiation (Figure S1). The early HFNC failure group had 50 (71.4%) and the late HFNC failure group had 20 patients (28.6%).

### 3.1. Clinical Characteristics and Outcomes

The clinical characteristics of all patients and both HFNC failure groups are presented in Table 1. The median age was 75 (IQR: 64–80), and the PaO_2_/FiO_2_ ratio at HFNC initiation was 151 (IQR: 93–248). The SOFA score was significantly higher in the early HFNC failure group than in the late HFNC failure group (5 [IQR: 2–9] vs. 3 [IQR: 1–4], *p* = 0.035). The duration of fever was significantly longer in the late failure group (4 days [IQR: 1–8 days] vs. 10.5 days [6–17.5], *p* = 0.004). There was no statistical difference in age, time from symptom to admission, chest radiographic findings, and treatments.

Overall in-hospital mortality was 45.7% (32 of 70), and the late failure group had a higher mortality rate than the early failure group (38.0% vs. 65.0%, *p* = 0.041). The duration of mechanical ventilation, length of hospital stay, and proportion of tracheostomy were not significantly different between the two groups.

### 3.2. Changes in ROX Index and SpO_2_/FiO_2_ Ratio

**Figure 2:**
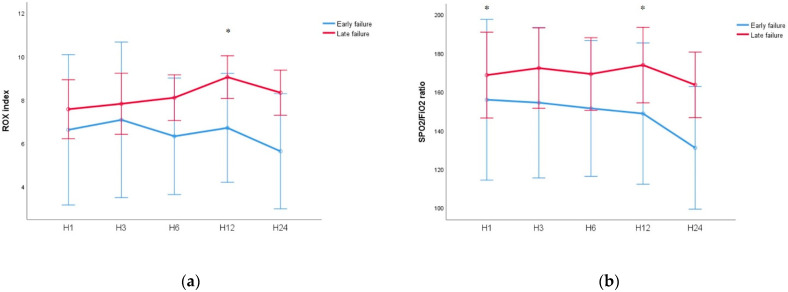
Changes in ROX index and SpO_2_/FiO_2_ ratio within 24 h from the HFNC initiation (**a**) ROX index (**b**) SpO_2_/FiO_2_ ratio. * *p* < 0.005 between groups. ROX = pulse oximetry/fraction of inspired oxygen/respiratory rate; SpO_2_ = percutaneous oxygen saturation; FiO_2_ = fraction of inspired oxygen; and HFNC = high-flow nasal cannula.

**Figure 3:**
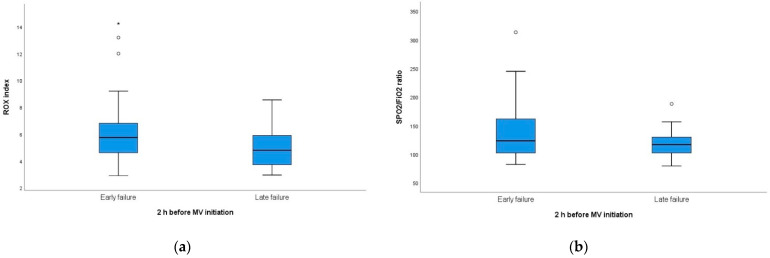
Comparison of ROX index and SpO_2_/FiO_2_ ratio before 2 h of intubation (**a**) ROX index, 5.74 (IQR: 4.58–6.98) in early failure group and 4.80 (IQR: 3.67–5.97) in late failure group, respectively (*p* = 0.040). (**b**) SpO_2_/FiO_2_ ratio, 125 (103–161) in early failure group and 116 (102–130) in late failure group, respectively (*p* = 0.134). ROX = pulse oximetry/fraction of inspired oxygen/respiratory rate; FiO_2_ = fraction of inspired oxygen; and SpO_2_ = percutaneous oxygen saturation.

The initial ROX index and SpO_2_/FiO_2_ ratio (at one hour of HFNC initiation) showed a tendency to be low in the early failure group (Table S1). Within 24 h of HFNC initiation, this tendency of the ROX index was maintained consistently (Figure 2). Within 24 h of HFNC initiation, the ROX index as well as SpO_2_/FiO_2_ ratio showed a tendency to be low in the early failure group (Figure 2). The ROX index at 12 h was 6.46 (IQR: 5.40–9.06) in the early failure group and 9.05 (IQR: 8.08–10.11, *p* = 0.017) in the late failure group. However, before two hours of intubation, the ROX index of the late failure group was lower than that of the early failure group (5.74 [IQR: 4.58–6.98] vs. 4.80 [IQR: 3.67–5.97], *p* = 0.040; Figure 3).

### 3.3. Predictive Factors of Mortality in Mechanically Ventilated COVID-19 Patients

The results of the univariate and multivariable logistic regression analyses for in-hospital mortality are shown in Table 2. In the univariate analysis, age ≥ 70 years (odds ratio [OR], 5.353 [95% CI: 1.793–15.985], *p* = 0.003), CCI ≥ 3 (OR, 6.304 [95% CI: 1.626–24.441], *p* = 0.008), SOFA score ≥ 3 (OR, 3.514 [95% CI: 1.287–9.593)], *p* = 0.014), neuromuscular blocker use (OR, 0.290 [95% CI: 0.099–0.853], *p* = 0.024), and late HFNC failure (OR, 3.030 [95% CI: 1.027–8.939], *p* = 0.045) were associated with mortality.

In the multivariable analysis, CCI ≥ 3 (OR 5.381 [95% CI: 1.179–24.559], *p* = 0.030), SOFA score ≥ 3 (OR 5.040 [95% CI: 1.344–18.899], *p* = 0.016), and late HFNC failure (OR 4.757 [95% CI: 1.118–20.236], *p* = 0.035) were independent predictive factors for in-hospital mortality.

The ROC curves of the scoring systems for in-hospital mortality are shown in Figure S2. The Area Under the Receiver Operating Characteristics (AUROCs) for the CURB-65, CCI, SOFA score, and APACHE II score were 0.590 (95% CI: 0.455–0.724), 0.719 (95% CI: 0.600–0.837), 0.593 (95% CI: 0.457–0.728), and 0.660 (95% CI: 0.532–0.788), respectively.

## 4. Discussion

In this multicenter study, we investigated the mortality rate and related factors in COVID-19 patients who were escalated to invasive mechanical ventilation after HFNC application. As this cohort comprised older patients, the in-hospital mortality rate was as high as 46%. Patients who underwent intubation more than two days after application of HFNC had a significantly higher mortality rate than those who received intubation within two days of HFNC. The late failure group had better oxygenation indices within 24 h of hospitalization than the early failure group; however, the ROX index before two hours of intubation was lower in the late failure group. In the multivariate analysis, the CCI, SOFA score, and intubation after 2 days of HFNC application were independent risk factors for in-hospital mortality.

Whether the intubation timing is associated with mortality in COVID-19 patients is unclear. Zirpe et al. reported that the mortality rates of patients who received intubation after 48 h and within 48 h of ICU admission were 77.7% and 60%, respectively [16]. On one hand, in a multicenter study with 755 mechanically ventilated patients, the time between admissions to intubation was significantly associated with mortality [17]; the result of the study demonstrated that the risk of death increased 1.03 times when intubation was delayed by one day. On the other hand, some recent studies reported that there is no association between time from ICU admission to intubation and mortality in COVID-19 patients [18,19]. A meta-analysis that included 8944 critically ill COVID-19 patients demonstrated that there was no statistical difference in mortality between patients who received early and late intubation (45.4% vs. 39.1%; RR, 1.07 [95% CI 0.99–1.15], *p* = 0.08) [19]. They argued that the decrease in the percentage of mechanical ventilation over time was a result of physicians avoiding mechanical ventilation as much as possible because of the assumption that the timing of intubation has no effect on mortality.

Many patients with severe COVID-19 do not present with severe dyspnea during profound hypoxemia, which is known as happy hypoxemia [20]. Although there is a possibility of sudden acute exacerbation, recovery of respiratory distress may be expected if the hyper-inflammation phase passes by [21]. Physicians caring for COVID-19 patients are struggling with the choice between the wait-and-see strategy and starting mechanical ventilation in patients with severe hypoxemia who seem comfortable. In this respect, our results provide important evidence. Patients whose respiratory distress gradually worsened after two days of HFNC initiation were intubated after aggravation of hypoxemia, compared to the patients who initially presented with severe hypoxemia. It is important to determine whether respiratory distress can be endured with HFNC rather than time from HFNC to intubation. We suggest that delayed intubation may affect mortality if the threshold is exceeded. Therefore, close observation of clinical parameters such as the ROX index is necessary in order to apply mechanical ventilation before the acute deterioration of respiratory failure.

Consistent with our findings, previous studies reported that the mortality rates of mechanically ventilated COVID-19 patients ranged from 38% to 45% [22,23,24]. The mortality increased exponentially with age [23,24]. In our cohort, which included relatively old patients with a median age of 75 years, the CCI and SOFA scores were independent risk factors for mortality. Our analysis indicates that comorbidities and organ dysfunction are risk factors for older patients who are mechanically ventilated. It has been known that comorbidities are important prognostic factors for COVID-19 patients. In a meta-analysis, CCI ≥ 3 was significantly associated with mortality (HR, 1.77 [1.68–1.86], *p* < 0.001) in a group of hospitalized COVID-19 patients [25]. In our study, the cohort of mechanically ventilated patients with a CCI ≥ 3 had an approximately 5.38-fold higher mortality risk than the others. Gao et al. reported that in-hospital death was associated with comorbidities (HR, 6.455, *p* = 0.007) and SOFA score on admission (HR 1.171, *p* = 0.033) in in-patients with severe COVID-19 [26]. In our study, a cohort of mechanically ventilated patients with a SOFA score of ≥3 had an approximately 5.04-fold higher mortality than the others. Raschke et al. reported that the accuracy of the SOFA score in predicting the risk of mortality in mechanically ventilated COVID-19 patients was not acceptable, as the AUC for the SOFA score was 0.59 [27]. However, the AUC of the CCI was 0.72, indicating that in mechanically ventilated older COVID-19 patients, the CCI can be useful for predicting mortality.

There are some limitations to this study. First, because of the retrospective study design, there were some missing values for respiratory parameters such as respiratory rate or ROX index. Second, this study included a relatively small number of patients. However, during the study period, the number of COVID-19 patients was relatively small in Korea. Nonetheless, our multicenter study enrolled as many severely ill patients as possible in seven referral hospitals for representativeness. Third, owing to the lack of standardized protocol for intubation, the timing of escalation from HFNC to mechanical ventilation would vary at each hospital. However, the intensivists at the hospitals may have reached a consensus on airway management because they had received the same critical care training at a center. Fourth, there were no data on the cause of death. Therefore, we do not know the association between late failure and death. In a study of the autopsy data of severe COVID-19 patients, the common causes of death were septic shock, multiorgan failure, or diffuse alveolar damage [28]. There is a concern regarding self-inflicted lung injury from the sustained high respiratory effort in spontaneously breathing patients [29]. A computational modeling study showed that the forces generated by increased inspiratory effort in acute hypoxic respiratory failure from COVID-19 are comparable to those involved in ventilator-induced lung injury [30]. Therefore, further prospective studies are needed to determine whether late failure causes diffuse alveolar damage.

## 5. Conclusions

Our data suggest that late failure of HFNC is associated with higher mortality in COVID-19 patients with acute respiratory failure compared with early failure. Patients whose respiratory distress gradually worsened after two days of HFNC initiation were intubated after aggravation of hypoxemia, compared to the patients with initially severe hypoxemia. Patients should be carefully monitored to determine whether a wait-and-see strategy is beneficial for severe COVID-19 patients, considering that delayed intubation can worsen the prognosis.

## Figures and Tables

**Table 1:** Baseline characteristics of COVID-19 patients treated with mechanical ventilation.

Variables	All Patients (*n* = 70)	Early Failure (*n* = 50)	Late Failure (*n* = 20)	*p* Value
Age (years)	75 (64–80)	71 (63–79)	77 (69–81)	0.176
Male (%)	41 (58.6)	30 (60.0)	11 (55.0)	0.701
Smoking (%)	12 (17.1)	8 (16.0)	4 (20.0)	0.732
Symptoms at admission (%)	69 (98.6)	50 (72.5)	19 (27.5)	0.286
Time from symptom to admission (days)	4 (2–7)	5.5 (2–7)	4 (2–6)	0.350
Time from HFNC initiation to intubation (hours)	11.3 (2.0–46.7)	4.1 (1.1–13.5)	70.9 (54.4–145.4)	<0.001
Body mass index (kg/m^2^)	25.4 (21.8–27.8)	25.5 (22.4–28.0)	24.4 (21.7–27.7)	0.447
Scoring systems				
CURB-65	2 (1–2)	1.5 (1–2)	2 (1–2)	0.731
SOFA score	4 (2–7)	5 (2–9)	3 (1–4)	0.035
APACHE II score	11 (8–13)	11 (8–14)	11 (8–13)	0.575
Charlson Comorbidity Index	4 (2–4)	4 (2–4)	4 (3–5)	0.181
Comorbidity (%)				
Hypertension	47 (67.1)	34 (68.0)	13 (65.0)	0.809
Diabetes	24 (34.3)	17 (34.0)	7 (35.0)	0.937
Chronic lung disease	6 (8.6)	3 (6.0)	3 (15.0)	0.343
Chronic kidney disease	3 (4.3)	2 (4.0)	1 (5.0)	1.000
Chronic liver disease	3 (4.3)	3 (6.0)	0 (0.0)	0.552
Cardiovascular disease	5 (7.1)	2 (4.0)	3 (15.0)	0.137
Neurologic disease	2 (2.9)	1 (2.0)	1 (5.0)	0.493
Malignancy	6 (8.6)	6 (12.0)	0 (0.0)	0.173
Vital signs				
Systolic blood pressure (mmHg)	135 (123–151)	133 (124–155)	136 (118–149)	0.640
Diastolic blood pressure (mmHg)	78 (67–90)	78.5 (68–90)	75 (64–84)	0.451
Heart rate (/min)	88.5 (75–98.5)	88 (74–101)	89.5 (77.5–98)	0.891
Respiratory rate (/min)	20 (20–24)	22 (20–25)	21.75 (18.5–20)	0.057
Body temperature (°C)	36.7 (36.4–37.4)	36.7 (36.4–37.6)	36.8 (36.5–37.3)	0.881
Oxygen saturation (%)	95 (90–97)	95 (89–97)	95.5 (91.5–98)	0.502
Glasgow Coma Scale	15 (15–15)	15 (15–15)	15 (15–15)	0.991
Duration of fever (days)	6 (1–11)	4 (1–8)	10.5 (6–17.5)	0.004
PaO_2_/FiO_2_ at HFNC initiation	151 (93–248)	154 (88–234)	141 (95–268)	0.943
Laboratory findings				
White blood cells (×10^9^/L)	6.5 (4.9–10.2)	7.0 (5.0–12.0)	5.9 (4.6–7.3)	0.092
Lymphocytes (×10^9^/L)	0.75 (0.38–1.08)	0.72 (0.38–1.15)	0.81 (0.39–0.97)	0.866
Protein (g/dL)	6.4 (6.0–6.8)	6.4 (6.0–6.9)	6.4 (6.1–6.8)	0.887
Creatinine (mg/dL)	0.80 (0.67–1.06)	0.79 (0.60–1.10)	0.87 (0.73–1.00)	0.320
C-reactive protein (mg/dL)	10.3 (6.0–18.2)	11.0 (6.6–18.8)	6.6 (2.6–12.8)	0.008
Chest x-ray (%)				0.065
Normal or unilateral	11 (15.7)	5 (10.0)	6 (14.0)	
Bilateral or multifocal	59 (84.3)	45 (90.0)	14 (70.0)	
Treatment (%)				
Remdesivir	28 (40.0)	20 (71.4)	8 (40.0)	1.000
Antibiotics	43 (61.4)	33 (66.0)	10 (50.0)	0.214
Vasopressor	33 (47.1)	25 (50.0)	8 (40.0)	0.449
Continuous renal replacement therapy	10 (14.3)	7 (14.0)	3 (15.0)	1.000
Corticosteroid	67 (95.7)	47 (94.0)	20 (100.0)	0.552
Neuromuscular blocker	49 (70.0)	38 (76.0)	11 (55.0)	0.083
Prone positioning	9 (12.9)	6 (12.0)	3 (15.0)	0.708
Outcomes				
In-hospital mortality (%)	32 (45.7)	19 (38.0)	13 (65.0)	0.041
Duration of mechanical ventilation (days)	15 (9–31)	15 (9–28.5)	11 (6–38)	0.458
Length of hospital stay (days)	22 (16–31)	27.5 (21–48)	27.5 (17–62)	0.948
Tracheostomy (%)	24 (35.8)	17 (34.7)	7 (38.9)	0.751

Values expressed as median (interquartile range) or n (%). COVID-19 = coronavirus disease 2019; HFNC = high-flow nasal cannula; CURB-65 = Confusion, Urea nitrogen, Respiratory rate, Blood pressure, 65 years of age and older; SOFA = Sequential Organ Failure Assessment; APACHE = Acute Physiology and Chronic Health Evaluation; PaO_2_ = partial pressure of oxygen; and FiO_2_ = fraction of inspired oxygen.

**Table 2:** Univariate and multivariate analyses of predictive factors for mortality.

Variables	Univariate Analysis		Multivariable Analysis	*p* Value
	OR (95% CI)	*p* value	OR (95% CI) *	
Age		0.003		
<70	Reference			
≥70	5.353 (1.793–15.985)			
Sex		0.184		
Female	Reference			
Male	0.520 (0.198–1.364)			
Smoking	0.820 (0.233–2.886)	0.757		
Body mass index	1.003 (0.892–1.127)	0.961		
Charlson Comorbidity Index		0.008		0.030
<3	Reference		Reference	
≥3	6.304 (1.626–24.441)		5.381 (1.179–24.559)	
CURB-65		0.091		
<2	Reference			
≥2	2.292 (0.875–6.002)			
SOFA score		0.014		0.016
<3	Reference		Reference	
≥3	3.514 (1.287–9.593)		5.040 (1.344–18.899)	
APACHE II score		0.074		
<10	Reference			
≥10	2.444 (0.916–6.526)			
Duration of fever	1.015 (0.977–1.054)	0.452		
Hypertension	0.679 (0.249–1.849)	0.449		
Diabetes	1.007 (0.374–2.713)	0.988		
Remdesivir	2.167 (0.818–5.737)	0.120		
Vasopressor	1.971 (0.759–5.120)	0.163		
Corticosteroid	0.405 (0.035–4.690)	0.470		
Neuromuscular blocker	0.290 (0.099–0.853)	0.024		
Time from HFNC initiation to intubation		0.045		0.035
<48 h	Reference		Reference	
≥48 h	3.030 (1.027–8.939)		4.757 (1.118–20.236)	
ROX index before intubation	0.799 (0.614–1.039)	0.094		
Chest X-ray		0.985		
Normal or unilateral	Reference			
Bilateral or multifocal	1.012 (0.278–3.688)			

* The clinical variables entered into the model were age, Charlson Comorbidity Index, CURB-65, SOFA score, APACHE II score, corticosteroid, neuromuscular blocker, time from HFNC initiation to intubation, and ROX index before 2 h of intubation. OR = odds ratio; CI = confidence interval; CURB-65 = Confusion, Urea nitrogen, Respiratory rate, Blood pressure, 65 years of age and older; SOFA = Sequential Organ Failure Assessment; APACHE = Acute Physiology and Chronic Health Evaluation; HFNC = high-flow nasal cannula; and ROX = pulse oximetry/fraction of inspired oxygen/respiratory rate.

## Data Availability

The datasets used and analyzed during the current study are available from the corresponding author on reasonable request.

## Supplementary Materials

The following are available online at https://www.mdpi.com/article/10.3390/jpm11100989/s1, Figure S1: Number of HFNC failure per day. HFNC = high-flow nasal cannula; and MV = mechanical ventilation, Figure S2: ROC curves of scoring systems for in-hospital mortality. AUROCs for CURB-65, CCI, SOFA score and APACHE II score were 0.590 (95% CI: 0.455–0.724), 0.719 (95% CI: 0.600–0.837), 0.593 (95% CI: 0.457–0.728), and 0.660 (95% CI: 0.532–0.788), respectively. AUROC = area under the receiver operating characteristic curve; CURB-65 = Confusion, Urea nitrogen, Respiratory rate, Blood pressure, 65 years of age and older; SOFA = Sequential Organ Failure Assessment; APACHE = Acute Physiology and Chronic Health Evaluation; CI = confidence interval, Table S1: Changes in respiratory variables within 24 h from the HFNC initiation.
