# Supplementary material for: Late Failure of High-Flow Nasal Cannula May Be Associated with High Mortality in COVID-19 Patients: A Multicenter Retrospective Study in the Republic of Korea
Source: J Pers Med. 2021 Sep 30;11(10):989. doi: 10.3390/jpm11100989 (PMC8540888; doi:10.3390/jpm11100989)
Supplement: Supplementary file 1 [file jpm-11-00989-s001.zip › Table S1.pdf]

Supplementary Table S1. Changes in respiratory variables within 24 hours from the HFNC initiation

Variables	All patients (n=70)	Early failure (n=50)	Late failure (n=20)	P value
ROX index				
1 h (n=59)	6.85 (5.40–8.43)	6.43 (5.33–8.10)	8.17 (6.13–10.56)	0.066
3 h (n=44)	6.96 (5.73–8.58)	6.60 (5.24–8.36)	7.25 (6.34–9.10)	0.338
6 h (n=37)	7.73 (5.85–9.75)	7.17 (5.32–9.75)	8.08 (7.51–9.75)	0.158
12 h (n=33)	8.64 (6.46–10.06)	6.46 (5.40–9.06)	9.05 (8.08–10.11)	0.017
24 h (n=24)	7.53 (6.25–9.61)	6.09 (5.13–8.89)	7.62 (7.07–10.43)	0.208
SpO_2/FiO_2				
1 h (n=67)	160 (120–186)	157 (118–174)	169 (148–222)	0.032
3 h (n=48)	158 (138–190)	151 (119–166)	163 (156–197)	0.053
6 h (n=39)	160 (140–190)	155 (124–172)	173 (160–194)	0.020
12 h (n=34)	163 (135–193)	139 (121–173)	182 (158–200)	0.051
24 h (n=24)	158 (140–192)	140 (110–207)	158 (143–192)	0.331
FiO_2				
1 h (n=67)	60 (50–80)	60 (50–80)	55 (40– 62.5)	0.027
3 h (n=49)	60 (50–75)	60 (60–80)	60 (50–60)	0.035
6 h (n=39)	60 (50–70)	60 (57.5–80)	55 (50–60)	0.035
12 h (n=34)	52.5 (42.5–67.5)	70 (50–80)	50 (50–60)	0.979
24 h (n=24)	60 (50–70)	70 (50–87.5)	60 (50–70)	0.208

Values expressed as median (interquartile range).

HFNC=high-flow nasal cannula; ROX=pulse oximetry/fraction of inspired oxygen/respiratory rate; SpO_2=percutaneous oxygen saturation; and FiO_2=fraction of inspired oxygen.
