# Supplementary figures and images for: Late Failure of High-Flow Nasal Cannula May Be Associated with High Mortality in COVID-19 Patients: A Multicenter Retrospective Study in the Republic of Korea
Source: J Pers Med. 2021 Sep 30;11(10):989. doi: 10.3390/jpm11100989 (PMC8540888; doi:10.3390/jpm11100989)

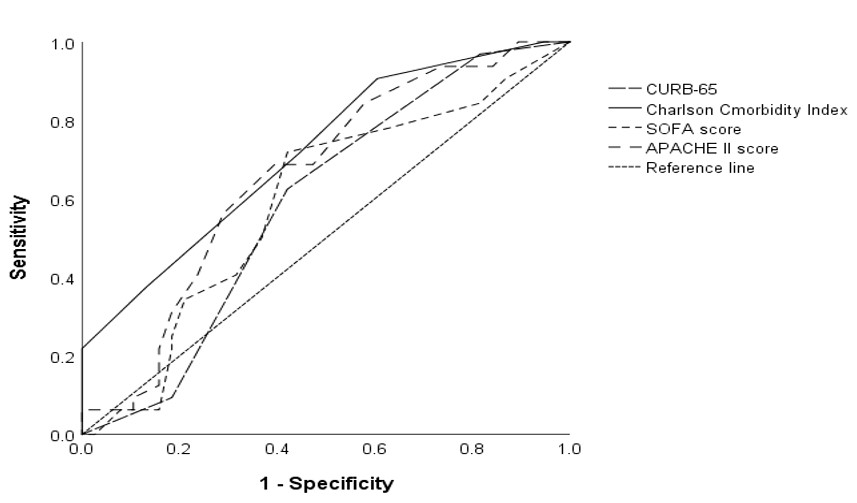

Supplement: Supplementary file 1 [file jpm-11-00989-s001.zip › FigS2.jpg]

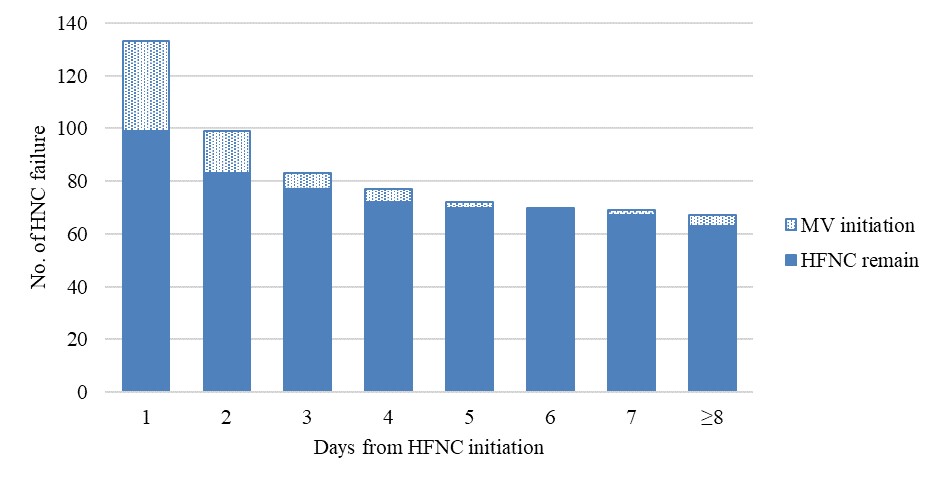

Supplement: Supplementary file 1 [file jpm-11-00989-s001.zip › FigS1.jpg]
